# Assessing Diagnostic Performance of Molecular Culture for Neonatal Sepsis: Protocol of the CHAMPIONS Study

**DOI:** 10.3390/diagnostics14171930

**Published:** 2024-09-01

**Authors:** Jip Groen, Martijn van der Kuip, Dries Budding, Martine P. Bos, Marc A. Benninga, Hendrik J. Niemarkt, Tim G. J. de Meij

**Affiliations:** 1 Department of Pediatric Gastroenterology, Amsterdam University Medical Center,1105 AZ Amsterdam, The Netherlands; m.a.benninga@amsterdamumc.nl; 2 Amsterdam Reproduction and Development Research Institute, 1105 AZ Amsterdam, The Netherlands; 3 Gastroenterology and Hepatology, Amsterdam Gastroenterology Endocrinology Metabolism, Amsterdam University Medical Center, University of Amsterdam, Meibergdreef 9, 1105 AZ Amsterdam, The Netherlands; 4 Department of Pediatric Infectious Diseases, Rheumatology and Immunology, Amsterdam University Medical Center, 1105 AZ Amsterdam, The Netherlands; m.vanderkuip@amsterdamumc.nl; 5 Inbiome, 1098 XG Amsterdam, The Netherlands; dries.budding@inbiome.com (D.B.); martine.bos@inbiome.com (M.P.B.); 6 Maxima Medical Center, Department of Neonatology, 5504 DB Veldhoven, The Netherlands; hendrik.niemarkt@mmc.nl

**Keywords:** neonatal, sepsis, diagnostic, culture, antibiotic, molecular, stewardship, PCR, bacteria

## Abstract

Managing neonatal sepsis is challenging due to nonspecific clinical signs, hematological markers with poor accuracy, and a lengthy turnaround time for the identification of microorganisms. Delaying the initiation of antibiotics in truly infected infants can lead to severe morbidity and mortality. Therefore, decisions regarding empiric antibiotic treatment are risk stratified, which exposes many uninfected infants to antibiotics. This causes gut microbiota perturbation, unnecessary hospital admissions, and the generation of multi-resistant organisms. High-speed diagnostic assays could expedite discontinuation or avert the initiation of antibiotics in uninfected infants. This study will evaluate the diagnostic performance of molecular culture (MC), a rapid broad-range PCR-based bacterial profiling technique, for diagnosing neonatal sepsis in infants below 90 days old. A multi-center prospective observational cohort study will include infants evaluated for early and late-onset sepsis. Routine evaluation for suspected sepsis includes microbiological cultures of blood. Additionally, blood for MC will be collected. For early-onset sepsis, umbilical cord blood may be used alternatively. Primary outcome is the agreement between MC and conventional blood culture results. Secondary outcome is the agreement of both assays with clinical sepsis using four different, commonly used definitions. Faster diagnostic pathways for sepsis may reduce antibiotic exposure time. Broad-range molecular assays may identify pathogens undetectable by conventional methods. Employment of umbilical cord blood samples for early-onset sepsis diagnosis can resolve challenges in collecting adequate blood volume and could further expedite treatment decisions.

## 1. Introduction

Management of neonatal sepsis is a commonly debated topic in the pediatric literature. Newborn infants possess a tolerogenic adaptive immune system and an immature innate immune system, which may contribute to poor defensive mechanisms against external pathogens [1,2], resulting in an increased risk of infection and sepsis. Accepting risks to the morbidity and mortality of these infants is culturally less acceptable. In the last decades, these risks have been mitigated by antibiotic prophylaxis for mother and child based on risk stratification. However, enhancing differentiation between truly infected and uninfected infants is a challenge. From a clinical perspective, this is complex, as symptoms can be nonspecific [3] and hematological indices lack accuracy for inflammation or infection [4]. Therefore, overtreatment of many is accepted to limit morbidity and mortality. This is reflected in data showing that up to a 95-fold number of infants are treated for suspected early-onset sepsis (EOS) [5,6], for every case of culture-proven EOS, which occurs in 1–4 per 1000 live-born infants [7,8,9,10]. For late-onset sepsis (LOS), this discrepancy is less profound, with up to a 20-fold number of infants treated for suspected LOS in cohorts of preterm infants below 32 weeks gestational age [6,11].

Whereas EOS, commonly defined as occurring <72 h of birth, is assumed to result mostly from vertical transmission of pathogens from mother to child, LOS, commonly defined as occurring >72 h and <3 months of age, is considered the result of horizontal transmission of pathogens [3,12]. Preterm infants are at increased risk of developing both forms due to frequent use of indwelling catheters, extended clinical admissions, and a more profound immaturity of host immune response, co-existing with gut barrier immaturity that may precede hematogenic translocation of bacteria residing in the gut [13,14]. The distinction in mode of transmission between EOS and LOS is supported by an epidemiological pattern of causative pathogens, with EOS episodes most frequently caused by *Escherichia coli* and *Streptococcus agalactiae* as prevalent commensals in the maternal birth tract, largely replaced by coagulase-negative staphylococci and *Staphylococcus aureus* for LOS [11,15,16]. Both entities share communal challenges in management and the risk of neurocognitive disability when adequate treatment is delayed [17,18].

As knowledge regarding the health effects of early-life antibiotic exposure increases, employing strategies to safely reduce unnecessary treatment becomes increasingly important. Evidence on the short-term health effects of antibiotic exposure in early life shows increased incidences of diarrhea [19], delayed enteral feeding [20], and necrotizing enterocolitis [21,22,23,24], as well as the formation of multidrug-resistant microorganisms [25]. Furthermore, increasing evidence on long-term health outcomes has been accumulated showing higher incidences of allergic diseases [26,27,28,29,30,31,32], obesity [27,33,34,35], diabetes [27,36,37], functional gastrointestinal disorders [38], and inflammatory bowel disease [36,39,40,41] in those exposed to early-life antibiotics. Strategies to reduce antibiotic exposure may therefore have health and cost benefits that extend beyond previous perspectives.

In limiting antibiotic exposure, two separate approaches can be identified. Firstly, differentiation between truly infected and uninfected infants prior to empiric treatment initiation should be optimized. Secondly, the decision point at which empiric antibiotic treatment is either prolonged and potentially modified to narrow spectrum options or discontinued may be expedited. The first option may serve a reduction in the number of infants treated, and the second a reduction in total antibiotic exposure time. As the availability of conventional culture results frequently prompts treatment decisions regarding the choice of antibiotic and prolongation or discontinuation, a faster broad-range diagnostic assay with similar or enhanced test characteristics for pathogen identification may expedite these decisions and reduce time to antibiotic exposure.

Several molecular methods and modifications to conventional culture pipelines have already been deployed to expedite identification of microorganisms [42]. However, some of these techniques have been limited by small-range microbial panels, as is the case in some multiplex PCRs [43,44,45,46], expensive sequencing or interpretation traits in next generation sequencing techniques [47], or only marginal improvements in turnaround time compared to conventional culture. While the development of more stringent criteria for empiric treatment initiation at the first suspicion of neonatal sepsis has already resulted in significant and safe reductions of unnecessary antibiotic treatment courses [48], a lot can be gained from expediting the second decision point.

The molecular culture (MC) technique is an unrestricted PCR-based bacterial profiling assay that allows species-specific identification through capillary electrophoresis of amplified interspace segments, which are situated between the bacterial 16S and 23S rRNA genes. Interspace segments are ubiquitous in bacteria, display species-specific lengths, and are often present as multiple copies on the bacterial genome. The use of fluorescently labeled primers adds additional discriminatory value by separating various phyla (taxonomic ranks) of bacteria and aids in bacterial load assessment by displaying relative fluorescence units (RFU). This pipeline allows high-speed result generation in 4–6 h following sample acquisition under a hypothesis-free approach. Molecular culture has been tested across various sample types, including joint aspirates and abscesses [49,50], and shows a high level of agreement with conventional culture results. Moreover, it identified bacteria in clinically suspect cases when the conventional culture remained negative. Also, a small study was performed using MC on blood in a cohort of neonates (*n* = 39) suspected of EOS [51]. No conventional culture confirmed EOS cases were included, but one culture-negative case showed both clinical and biochemical signs of infection and a positive MC, indicating *E. faecalis*. Contaminant species were found in two MC analyses additional to conventional culture.

Increased knowledge on the perturbing effects of antibiotic overuse demand advances in the management of neonates suspected of sepsis. High-speed molecular assays for pathogen detection and identification may help to solve this issue. We therefore propose a diagnostic accuracy study of the performance of MC in a large cohort of neonates suspected of sepsis.

## 2. Materials and Methods

### 2.1. Study Design and Setting

A multi-center prospective observational cohort study will be performed in 11 centers across the Netherlands, in both level 2 and 3 NICUs, general maternity and neonatology wards, and the emergency room. Ethical clearance was acquired from the ethics board of the Amsterdam University Medical Center, file numbers NL84592.018.23 and W22_453#22.533. The Standards for Reporting Diagnostic Accuracy (STARD) 2015 checklist was utilized for reporting in this protocol and in future reports on the results [52]. No patient, parent, or public stakeholder has been involved in the study design of this protocol. However, when the results of this study legitimize an implementation study, we aim to involve these parties for future works.

### 2.2. Study Aim

Primary outcome is the agreement of molecular culture results with conventional culture results in suspected neonatal sepsis cases. Secondary outcome is the agreement of MC results with clinical sepsis, compared to the agreement of conventional culture results with clinical sepsis. Four separate definitions of clinical sepsis are prospectively outlined in this protocol (Table 1) to meet low- and high-risk criteria for clinical syndrome definitions and will all be tested for agreement with both MC and conventional culture results in cases where MC and conventional culture results are discordant. Agreement will be expressed using common measures to assess diagnostic accuracy, i.e., sensitivity, specificity, and positive and negative predictive values.

We hypothesize that test sensitivity as compared to conventional culture results will be above 90% on sample level (i.e., positivity and negativity), which was used for power analysis. We also expect that MC will detect true pathogens in a subset of cases with clinical suspicions of sepsis but with negative cultures, implying enhanced sensitivity for clinical sepsis. We hypothesize that MC on umbilical cord blood samples will have lower sensitivity and specificity for the conventional culture results for the primary outcome, given alternative blood collection sources that lead to varying contamination profiles. Lastly, we hypothesize that MC will yield more contamination or clinically irrelevant species since molecular methods do not differentiate between dead or live microorganisms.

### 2.3. Participant Screening, Inclusion, Data Collection

All infants <90 days of age who are evaluated for sepsis, including a conventional culture-based assay on blood, are eligible for participation and will be recruited consecutively. Inclusion start dates vary between participating centers in anticipation of the authorization of respective ethics boards. The first patient was enrolled on 27 July 2023. At the time of protocol submission, not all 11 centers have initiated patient recruitment, and no data analysis has been performed.

No restrictions for gestational age were set. In the case of suspected LOS, the study-related blood sample will be taken from the same source from which blood was collected for the conventional culture under aseptic conditions (i.e., venipuncture, intravenous cannulization, central venous catheter). In the case of suspected EOS, the study-related blood sample may be taken from the same source as conventional culture, or alternatively from the umbilical cord within 30 min after birth or from both, also under aseptic conditions. No additional phlebotomies or catheter placement procedures will be undertaken for the purposes of this study. The aim is to collect 1 mL of study-related blood for each participant, but if for any reason this is not feasible or does not comply with blood volume regulations for pediatric research [53], as may be the case for low birth weight (preterm) infants, smaller volumes may be collected and volume registration included as a variable. Parental or caregiver consent will be acquired from both parents and caregivers to finalize study participation.

Data collection is planned for after the performance of the reference standard test but before the index test. Clinical data and reference test results will not be available to the performers and readers of the index test.

### 2.4. Power Calculation

Based on the hypothesis that MC has 100% (lower CI 90%) sensitivity compared to conventional culture on a sample level (i.e., positivity and negativity of results), to reject the null hypothesis that sensitivity is below 90% with a power of 0.96, it is required to include at least 36 infants with a positive conventional blood culture. Based on previous literature, we expect that 2% of all infants undergoing sepsis evaluation for EOS will have a positive blood culture [5] and 10% of preterm infants (gestational age < 32 weeks) undergoing evaluation for LOS [11,54]. When assessing EOS and LOS separately, this would translate to respective estimated study populations of 1800 and 360 subjects. Since the power analysis relies on microbiological culture positivity, we aim to generate a mixed cohort of suspected EOS and LOS cases and publicize the initial results after enrolling 200 children to assess the initial culture positive cases and contamination rates, to warrant a larger sample size.

### 2.5. Sample Processing, Analysis, Interpretation of Results

**Figure 1:**
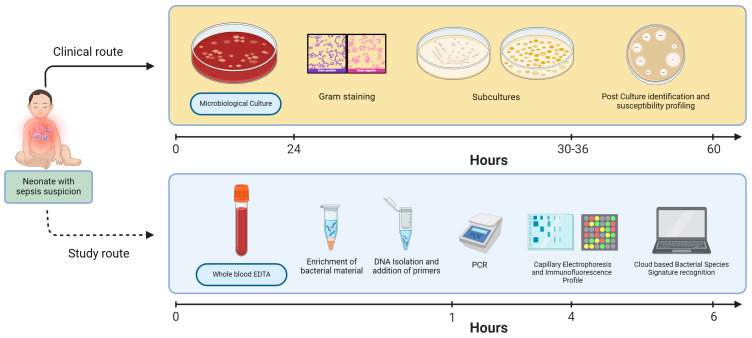
Patient study scheme. Upper panel; summarizes routine performance of microbiological culture assay (reference test); lower panel; summarizes study-related MC laboratory pipeline (index test).

After samples have been collected and pseudonymized, they undergo no further processing and are stored within 4 h at a temperature of −70 degrees C until further handling. MC performance is schematically outlined in Figure 1. Prior to DNA isolation, bacterial enrichment of blood samples will be performed by applying the Polaris protocol that involves selective lysis of human cells to support degradation of human genetic material, which allows enhanced detectability of bacterial material (Inbiome, Amsterdam, the Netherlands) [55]. MC will be performed as described previously in a published protocol by the manufacturer [50]. MC will be conducted using the molecular culture kit. In brief, the preprocessed blood sample will be mixed with 250 μL of shock buffer 1 and incubated at 95 °C for 10 min with continuous shaking at 800 rpm. Subsequently, 25 μL of shock buffer 2 will be added to the samples. One milliliter of EasyMag lysis buffer (bioMérieux, Durham, NC, USA) and 1 mL of AL buffer (Qiagen, Hilden, Germany) will then be incorporated prior to the extraction of DNA using the Specific A Protocol on the automated EMAG extraction system (bioMérieux, Marcy-l’Étoile, France). The DNA will be eluted in a final volume of 70 μL.

Two separate PCR reactions will be performed, each utilizing 10 μL of the extracted DNA. The first PCR will target the phyla Firmicutes, Actinobacteria, Fusobacteria, Verrucomicrobia, and Bacteroidetes, while the second PCR will target Proteobacteria and an internal amplification control. The resulting PCR products will be combined and analyzed for amplicon length and fluorescence intensity, measured as relative fluorescence units (RFU), using an ABI3500 fragment analyzer (ThermoFisher, Waltham, MA, USA). Data from the ABI3500 will be analyzed using Antoni, a Lab Cloud software system that matches amplicon lengths and fluorescence intensity to bacterial species through a dedicated database (Inbiome, Amsterdam, the Netherlands).

### 2.6. Reference Test

PEDS BacT/Alert (bioMérieux Inc., Durham, NC, USA) blood culture bottles will be inoculated with preferably 1 mL of infant blood. The inoculated bottles will subsequently be placed in the BacT/Alert microbial detection system (bioMérieux Inc.). This system maintains the bottles at 35 °C. Microorganism growth induces the production of carbon dioxide. A light-emitting diode (LED) projects light onto this sensor, and as more carbon dioxide is produced, the reflected light increases. The sensor continuously monitors and compares the carbon dioxide levels to the initial baseline. In the case where these levels exceed baseline, Gram staining and subcultures will be performed accordingly, followed by further post-culture identification methods appropriate to the initial results.

Performance of the reference test will be conducted by clinical microbiology services outside the scope of the research team. Small differences may exist in the pipeline of individual microbiology laboratories, as the sites included in this study all have separate facilities.

### 2.7. Definitions

Test agreement for primary outcome measure (to meet definition of agreement between conventional culture and MC, the following criteria should be met)

Sample level agreement

1. 1. Both test results should be either positive or negative

Species-level agreement

2. Both tests should indicate the same bacterial species
3. In case of discrepancies between the two tests in the amount of species cultured (i.e., mono- vs. polymicrobial results), both tests should at least share one bacterial species.

Test positive definitions
Positive conventional culture (reference test) (to meet the definition, the result should meet the following criteria)

1. Peripheral blood sample that has been cultured in at least 1 aerobic pediatric culture bottle (or in 1 aerobic and 1 anaerobic bottle) and yields a positive result.
2. For EOS, cultured coagulase-negative staphylococci (CoNS) will be considered contaminant organisms for neonates without indwelling catheters in situ prior to microbiological culture sampling. For LOS, CoNS may always be considered pathogenic.
3. Polymicrobial culture results will be considered for pathogenicity.

Positive molecular culture (index test) (to meet the definition, the result should meet the following criteria)

1. Umbilical cord blood sample collected <30 min postpartum OR peripherally/centrally collected blood sample that yields a positive result.
2. For EOS, cultured coagulase-negative staphylococci (CoNS) will be considered contaminant organisms for neonates without indwelling catheters in situ prior to microbiological sampling. For LOS, CoNS may always be considered pathogenic.
3. Polymicrobial culture results will be considered for pathogenicity.

Radiological indices will not be considered a standalone factor in defining clinical sepsis. Risk factors for EOS (such as prolonged rupture of membranes, maternal fever during labor, maternal GBS positivity, or amnionitis) will not be considered standalone factors in defining clinical sepsis.

The authors may apply post hoc exploratory definitions for clinical sepsis if the updated literature warrants this or otherwise new insights support this. Labeling of such definitions as “exploratory” will always be explicitly mentioned, as it forms a clear source of bias if not predefined.

### 2.8. Statistical Analysis

Baseline characteristics will be presented separately for suspected EOS and LOS cases. Also, baseline data will be presented separately for all included patients who meet specific clinical sepsis definitions (Table 1). Continuous data will be presented as means (standard deviations) or medians (interquartile range) depending on the data distribution. Categorical data will be presented as numbers (percentages). MC results will be cross-tabulated against conventional culture results on a sample basis (positive vs. negative results) and a species basis (similar species identification). Separate tables will be displayed comparing MC and conventional culture to clinical sepsis with all four prespecified clinical sepsis definitions (Table 1). The secondary outcome will only be assessed for cases with discordant MC and conventional culture results. Data on umbilical cord blood and peripheral blood will be reported separately for EOS. Statistical analyses will be performed in SPSS version 25.0 (IBM Corp. Released 2017. IBM SPSS Statistics for Windows, Version 25.0. Armonk, NY, USA: IBM Corp.).

## 3. Discussion

This study will assess the diagnostic accuracy of the MC for neonatal sepsis. There is a high demand for fast and broad-range pathogen-identifying assays to complement current diagnostic strategies. Given the large amount of antibiotic overtreatment of infants, juxtaposed with increasing knowledge regarding the disruptive effects of antibiotics, the need for assays with a high negative predictive value to exclude neonatal sepsis is underlined. Whereas the implementation of such assays may initially target expedited treatment discontinuation in well-appearing infants, secondary to that, watchful waiting may become acceptable for well-appearing infants prior to starting treatment. Assuming shorter turnaround times of MC and adequate diagnostic performance, the potential exists to improve antibiotic stewardship for this population with the primary focus of faster de-escalation or preventing unnecessary treatment.

The current study protocol will not assess the turnaround time of MC in clinical practice and the potential effect of its implementation on treatment decisions. Rather, it is primarily focused on assessing diagnostic performance, separate from diagnostic speed. When diagnostic performance is substantiated, future studies need to address the actual turnaround time of MC in clinical practice, as well as the effect of decision making with regards to faster de-escalation of antibiotic therapy in the case of test negativity and narrowing of antibiotic regimens in the case of positive MC. We hypothesize that, given sufficient diagnostic performance, implementation of MC may prove cost efficient based on reduction of hospital stay and through reducing the occurrence of health care outcomes associated with antibiotic exposure in early life.

The current pipeline for MC analysis does not allow for the generation of antibiotic susceptibility profiles. The employment of genetic material in MC may support implementation of detection of common resistance genes as a derivative of antibiotic susceptibility profiling, as has been done in a research context for several molecular methods [56,57,58]. However, experts believe that such pathways will not lead to valid alternatives for conventional susceptibility testing, as done by disk diffusion, broth microdilution, and agar dilution [59,60]. Pheno-molecular susceptibility testing may provide an answer to that issue in the future [61]. In the current MC protocol, conventional culture will thus remain necessary in true infections to guide antibiotic therapy. However, even without susceptibility testing, mere pathogen identification may already guide therapeutic choices and increase the prognosis of the infant, as can be the case for certain Gram-negative bacteria [62].

Given that MC would be deployed alongside conventional culture initially, two challenging scenarios may present. Firstly, MC results may return negative, and clinicians may decide to discontinue treatment based on this. Subsequently, conventional culture turns positive after empiric treatment has been discontinued. Given a true infection, clinical deterioration should occur, but at least this discrepancy should guide intensive monitoring, and at the very least, reinitiation of antibiotic therapy could be considered. Secondly, MC results may return positive and conventional culture negative. This could trigger one of two decisions: either broad-spectrum antibiotics are continued in the absence of conventional culture confirmation and susceptibility profiles, which is already a common issue in current practice [5,63]. Alternatively, clinicians may decide to discontinue antibiotics based on the notion that conventional culture has better specificity than MC. Moreover, this latter decision would only be made in the case of a reassuring clinical state that strongly reduces suspicion of infection.

As is reflected in a recent systematic review on definitions of neonatal sepsis [12], there is a great variety in the landscape of used diagnostic criteria. We emphasize that any definition of neonatal sepsis may yield specific critiques and find that the choice of variables remains contextual and personal. However, we will attempt to at least partially circumvent this issue by deploying multiple prospectively outlined definitions with the aim of transparently creating an optimal foundation for such a diagnostic accuracy study. This may solve issues in the case of discrepant MC and conventional culture results. As these discrepancies will certainly occur, it will be of particular interest to learn whether MC may provide enhanced sensitivity when tested against these various clinical definitions.

## Figures and Tables

**Table 1:** Sepsis definitions; to meet definition criteria for each separate risk category, patients should meet all criteria. For the moderate risk profile, EOS and LOS are defined differently.

**Low Risk**	**Moderate Risk EOS**	**Moderate Risk LOS**	**Severe or Septic Shock**
≥24 h of (empiric) antibiotic treatment following culture sampling or requirement to (re)initiate antibiotic treatment <3 days after initial evaluation following clinical deterioration and renewed or sustained suspected infection	≥24 h of (empiric) antibiotic treatment following culture sampling or requirement to (re)initiate antibiotic treatment <3 days after initial evaluation following clinical deterioration and renewed or sustained suspected infection	≥24 h of (empiric) antibiotic treatment following culture sampling or requirement to (re)initiate antibiotic treatment <3 days after initial evaluation following clinical deterioration and renewed or sustained suspected infection	≥24 h of (empiric) antibiotic treatment following culture sampling or requirement to (re)initiate antibiotic treatment <3 days after initial evaluation following clinical deterioration and renewed or sustained suspected infection
Highest C-reactive protein during the first 36 h after culture sampling ≥ 10 mg/L	Highest C-reactive protein during the first 36 h after culture sampling ≥ **20** mg/L	Highest C-reactive protein during the first 36 h after culture sampling ≥ **10** mg/L	
	≥1 of the following symptoms: Apnea, postpartum CPR, temperature instability (<36 or >38 degrees centigrade, respiratory distress, altered behavior, altered muscle tone, feeding difficulties, feeding intolerance, brady-/tachycardia, hypoxia, persistent pulmonary hypertension, jaundice <24 h post-partum, encephalopathy, coagulation abnormalities, acidosis	≥1 of the following symptoms: Apnea, postpartum CPR, temperature instability (<36 or >38 degrees centigrade, respiratory distress, altered behavior, altered muscle tone, feeding difficulties, feeding intolerance, brady-/tachycardia, hypoxia, persistent pulmonary hypertension, jaundice <24 h post-partum, encephalopathy, coagulation abnormalities, acidosis	At any point shortly before culture sampling or during antibiotic treatment following culture sampling and potentially relatable to infection ≥1 of the following events: a. Circulatory insufficiency requiring fluid resuscitation and/or inotropic support b. Respiratory insufficiency requiring mechanical ventilation c. Altered consciousness d. Seizures

## Data Availability

We intend to comply with future requests for datasets after anonymizing entries following local privacy regulations.
